# Metabolomic and lipidomic changes triggered by lipopolysaccharide-induced systemic inflammation in transgenic APdE9 mice

**DOI:** 10.1038/s41598-021-92602-4

**Published:** 2021-06-22

**Authors:** Elena Puris, Štěpán Kouřil, Lukáš Najdekr, Sanna Loppi, Paula Korhonen, Katja M. Kanninen, Tarja Malm, Jari Koistinaho, David Friedecký, Mikko Gynther

**Affiliations:** 1 School of Pharmacy, University of Eastern Finland, P.O. Box 1627, 70211 Kuopio, Finland; 2 Institute of Molecular and Translational Medicine, Palacký University Olomouc, Hněvotínská 5, 77900 Olomouc, Czech Republic; 3 Department of Clinical Biochemistry, University Hospital Olomouc, I.P. Pavlova 6, 77900 Olomouc, Czech Republic; 4 A.I. Virtanen Institute for Molecular Sciences, University of Eastern Finland, P.O. Box 1627, 70211 Kuopio, Finland; 5 Present Address: Institute of Pharmacy and Molecular Biotechnology, Ruprecht-Karls-University, Im Neuenheimer Feld 329, 69120 Heidelberg, Germany; 6 Present Address: Department of Immunobiology, University of Arizona, 1656 E Mabel Street, Tucson, AZ 85724-5221 USA; 7 Present Address: Neuroscience Center, Helsinki Institute for Life Science, University of Helsinki, Haartmaninkatu 8, 00290 Helsinki, Finland

**Keywords:** Lipidomics, Metabolomics

## Abstract

Peripheral infections followed by systemic inflammation may contribute to the onset of Alzheimer`s disease (AD) and accelerate the disease progression later in life. Yet, the impact of systemic inflammation on the plasma and brain tissue metabolome and lipidome in AD has not been investigated. In this study, targeted metabolomic and untargeted lipidomic profiling experiments were performed on the plasma, cortices, and hippocampi of wild-type (WT) mice and transgenic APdE9 mice after chronic lipopolysaccharide (LPS) treatment, as well as saline-treated APdE9 mice. The lipidome and the metabolome of these mice were compared to saline-treated WT animals. In the brain tissue of all three models, the lipidome was more influenced than the metabolome. The LPS-treated APdE9 mice had the highest number of changes in brain metabolic pathways with significant alterations in levels of lysine, myo-inositol, spermine, phosphocreatine, acylcarnitines and diacylglycerols, which were not observed in the saline-treated APdE9 mice. In the WT mice, the effect of the LPS administration on metabolome and lipidome was negligible. The study provided exciting information about the biochemical perturbations due to LPS-induced inflammation in the transgenic AD model, which can significantly enhance our understanding of the role of systemic inflammation in AD pathogenesis.

## Introduction

Alzheimer's disease (AD) is a progressive, incurable neurodegenerative disorder (ND) with the highest prevalence among all NDs^1^. The major neuropathological hallmarks of AD include the formation of extracellular deposits of fibrillar and amorphous amyloid-β peptide (Aβ) aggregates and intracellular neuronal aggregates of hyperphosphorylated tau forming neurofibrillary tangles (NFTs). Their formation in brain regions such as the entorhinal cortex, hippocampus, basal forebrain, and amygdala are associated with neuronal degeneration and synaptic loss, resulting in impaired learning and memory functions. Familial AD (fAD) caused by mutations in one of three genes involved in Aβ formation, namely, presenilin 1 and 2 (PSEN1 and PSEN2) and β-amyloid precursor protein (APP), can be diagnosed using genetic testing. In contrast, despite the extensive research, the mechanisms underlying the development and progression of late-onset or sporadic AD (sAD), which accounts for up to 95% of cases, are still unknown.

Several studies demonstrated that inflammatory response manifested by reactive microglia surrounding amyloid plaques may play an essential role in AD pathogenesis and occurs before the onset of clinical symptoms^2–4^. Whilst microglial reactions to acute injuries have initial beneficial aims^2,5^, the inability of microglia to clear the abnormally accumulating Aβ leads to chronic self-promoting neuroinflammation, which may be harmful to nervous tissue^6,7^. There is evidence that peripheral infections accompanied by systemic inflammation may contribute to the onset of sAD. For example, infections can cause AD-like pathological alterations, suggesting that infection-mediated changes in the brain may facilitate the susceptibility of developing AD later in life^8,9^. Resulting from infection, systemic inflammation can lead to neuroinflammation attributed to activated microglia, the release of numerous cytokines, and recruitment of peripheral immune cells into the brain^10^. Increasing epidemiological and animal studies have provided important evidence that systemic inflammatory conditions resulted from infections may be associated with increased AD risk and accelerate AD progression^10,11^. In wild-type (WT) mice, the systemic administration of a Gram-negative bacterial cell wall protein and an endotoxin lipopolysaccharide (LPS), a Toll-like receptor 4 (TLR-4) ligand, resulted in the peripheral stimulation of the synthesis of pro-inflammatory cytokines in the brain^12^. Besides, the systemic administration of LPS to APPswe transgenic mice resulted in increased APP expression, the intracellular accumulation of Aβ peptide, and elevated memory deficits^13^. Thus, infection and associated neuroinflammation may play crucial roles in AD development.

The present study aimed to investigate the effects of systemic inflammation in AD on the plasma and brain metabolome and lipidome. To achieve the goal, we induced systemic inflammation by chronic intraperitoneal (i.p.) injections of LPS in transgenic model APPswe/PS1dE9 (APdE9) mice^14^. The APdE9 mouse model overexpressing the Swedish mutation of APP and PSEN1 deleted in exon 9 have shown to reproduce several AD-related features, i.e. Aβ plaques, deficits in neuronal activity, mild neuritic abnormalities, the impairment of pre- and postsynaptic cholinergic transmission, and elevated mortality^15–19^. We compared the metabolome and lipidome in LPS-treated APdE9 mice (hereinafter referred to as the APdE9 plus LPS group) to age-matched saline-treated WT mice (hereinafter referred to as the WT control group). Additionally, the metabolome and lipidome of saline-treated APdE9 mice (hereinafter referred to as the APdE9 group) and WT mice with LPS treatment (hereinafter referred to the WT plus LPS group) were compared to age-matched saline-treated WT mice. Finally, the changes in metabolome and lipidome between the above mentioned models were compared. Two comprehensive analytical approaches were used to describe and understand pathobiochemical processes. Targeted metabolomics and untargeted lipidomics were applied to cover major metabolic pathways and to obtain a general overview of altered lipid classes and species.

## Results

The aim of the study was to compare the metabolome and lipidome in LPS-treated APdE9 mice, saline-treated WT mice and saline-treated APdE9 mice to age-matched saline-treated WT controls in order to investigate the effect of systemic inflammation in AD. We previously characterized a sixteen-month-old APdE9 mouse model in terms of increased formation of Aβ plagues and impairment in spatial learning and memory^19,20^. In the present study, the novel object recognition (NOR) test revealed decreased cognition, particularly recognition memory, in all three mouse models compared to WT controls (Supplementary information 1, Supplementary Fig. S1 online). In addition, LPS treatment affected locomotor exploratory activity of mice during the Open Field Spontaneous Movement Activity test as indicated by increased ambulatory distance and ambulatory time as compared to WT controls (Supplementary Fig. S2 online). No changes in locomotor exploratory activity were observed in the APdE9 mice or WT plus LPS group compared to WT controls.

The cortex and hippocampus analyses in three mouse models and the WT controls resulted in the detection of 153 metabolites by targeted metabolomics approach and 1435 features by the untargeted lipidomics approach (Figs. 3, 4, Supplementary Fig. S3, Supplementary Table S1, S2 online). In plasma of three mouse models and WT controls, 144 metabolites and 482 lipids were detected (Supplementary Fig. S3, Supplementary Table S3, S4 online).

**Figure 1:**
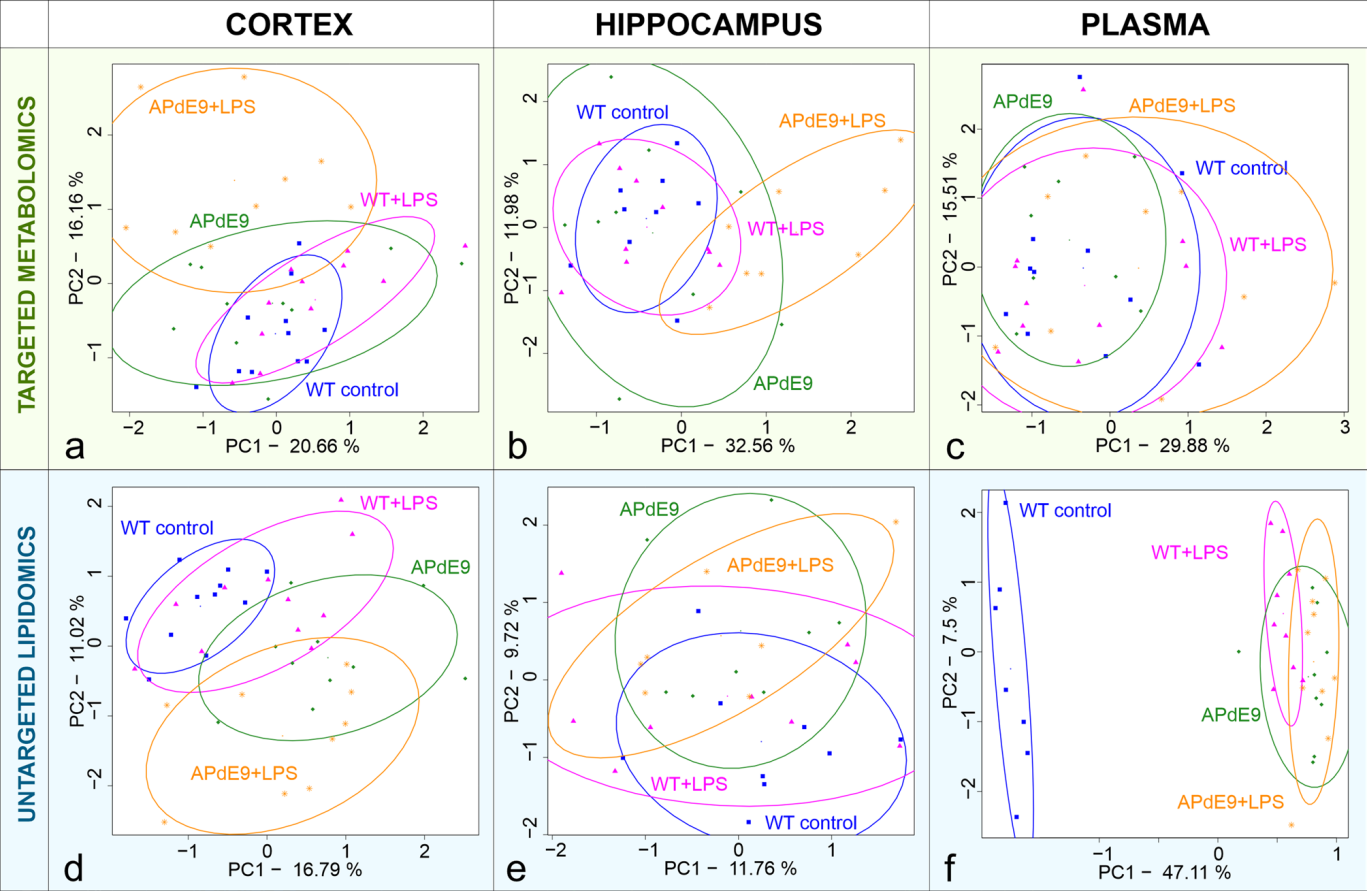
Principal component analysis (PCA) score plots for each model and analytical approach studied to compare global differences in the metabolomic (**a**–**c**) and lipid composition (**d**–**f**) of the hippocampus, cortex and plasma in all the groups that were studied.

From a global perspective, the overall differences and similarities in the hippocampal and cortical lipids and metabolites between all the groups were assessed using the unsupervised multivariate statistical method—principal component analysis, PCA (Fig. 1). The hippocampal and cortical metabolome of the WT plus LPS mice overlapped with the WT control group, whereas the APdE9 mice and the APdE9 plus LPS mice exhibited slightly better separation from the WT control group in the cortical and hippocampal metabolome (Fig. 1a,b) and lipidome (Fig. 1d,e). Comparing the plasmatic profiles of WT control and other groups of mice, the administration of LPS and/or APdE9 mutation had a little effect on polar metabolites (Fig. 1c) but a significant effect on the complex lipidome (Fig. 1f).

### Targeted metabolomic analysis of the brain tissue (cortex and hippocampus)

**Figure 2:**
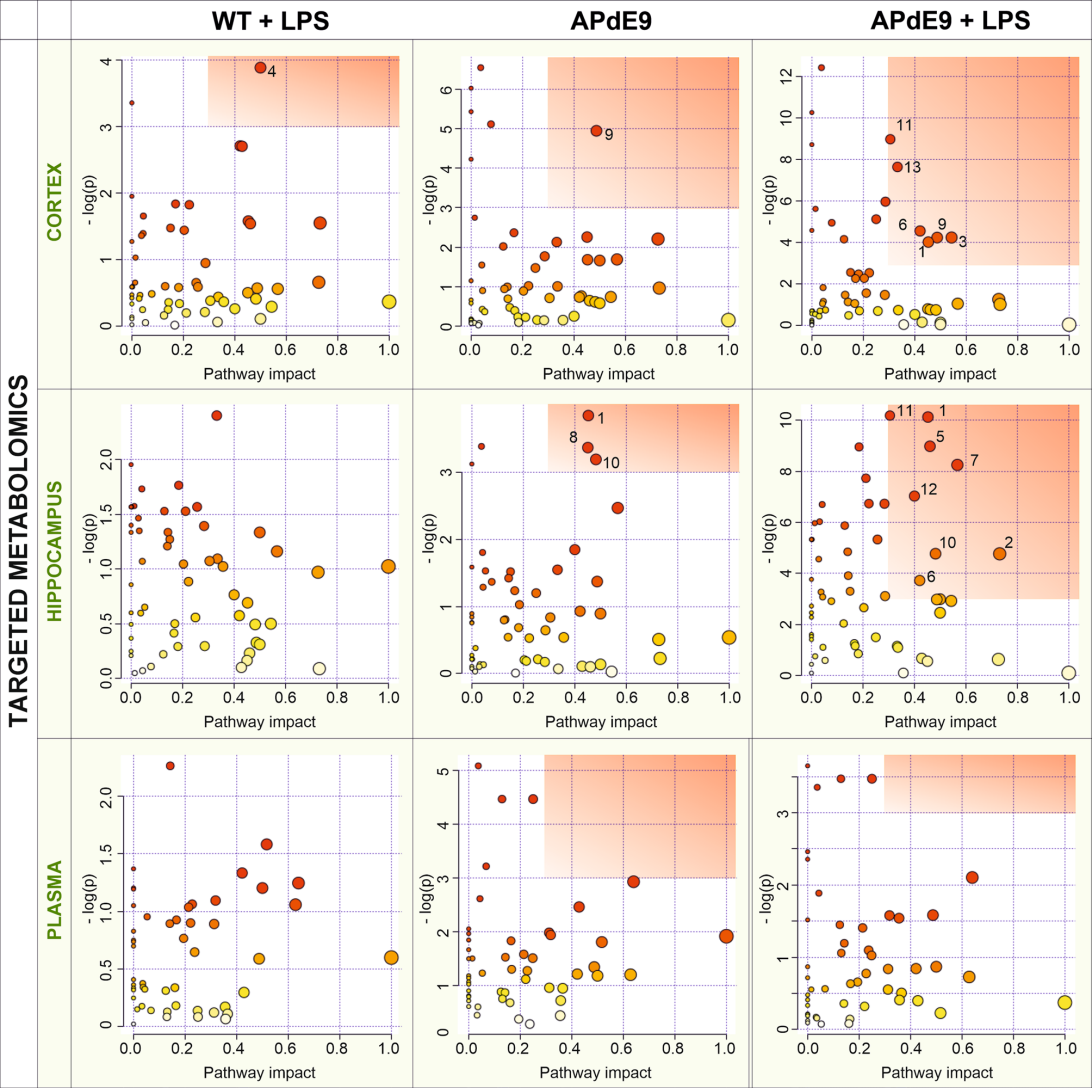
Biochemical pathway analysis (MetaboAnalyst 4.0, https://www.metaboanalyst.ca/) performed on targeted metabolomic data in the hippocampus, cortex and plasma of all groups studied versus the respective controls. The red rectangle highlights the most affected pathways with − log(*p* value) greater than 3 and a pathway impact greater than 0.3 for all graphs. Individual pathways are numbered as follows: 1—arginine and proline metabolism, 2—alanine, aspartate, and glutamate metabolism, 3—cysteine and methionine metabolism, 4—riboflavin metabolism, 5—nicotinate and nicotinamide metabolism, 6—pyrimidine metabolism, 7—β-alanine metabolism, 8—histidine metabolism, 9—carbohydrate metabolism, 10—arginine biosynthesis, 11—fatty acid degradation, 12—glutathione metabolism, 13—arachidonic acid metabolism.

The results of the biochemical pathway analysis (Fig. 2) were in accordance with the findings from the unsupervised PCAs described above. The LPS administration did not alter the cortical and hippocampal metabolome of the WT mice but induced additional changes in the cortical and hippocampal metabolome to those observed in the APdE9 mice. In both models, the APdE9 and APdE9 plus LPS mice, compared to WT control animals, cortical arginine and proline metabolism, arginine biosynthesis as well as hippocampal carbohydrate metabolism were affected (Fig. 2). The administration of LPS in the APdE9 mice provided the changes in the following biochemical pathways, which were additional to those observed in the APdE9 mice: cysteine and methionine metabolism, carbohydrate metabolism, arachidonic acid metabolism in the cortex; nicotinate and nicotinamide metabolism, β-alanine metabolism, glutathione metabolism and alanine, aspartate, and glutamate metabolism in the hippocampus; arginine and proline metabolism and fatty acid degradation in both the cortex and hippocampus (Fig. 2).

**Figure 3:**
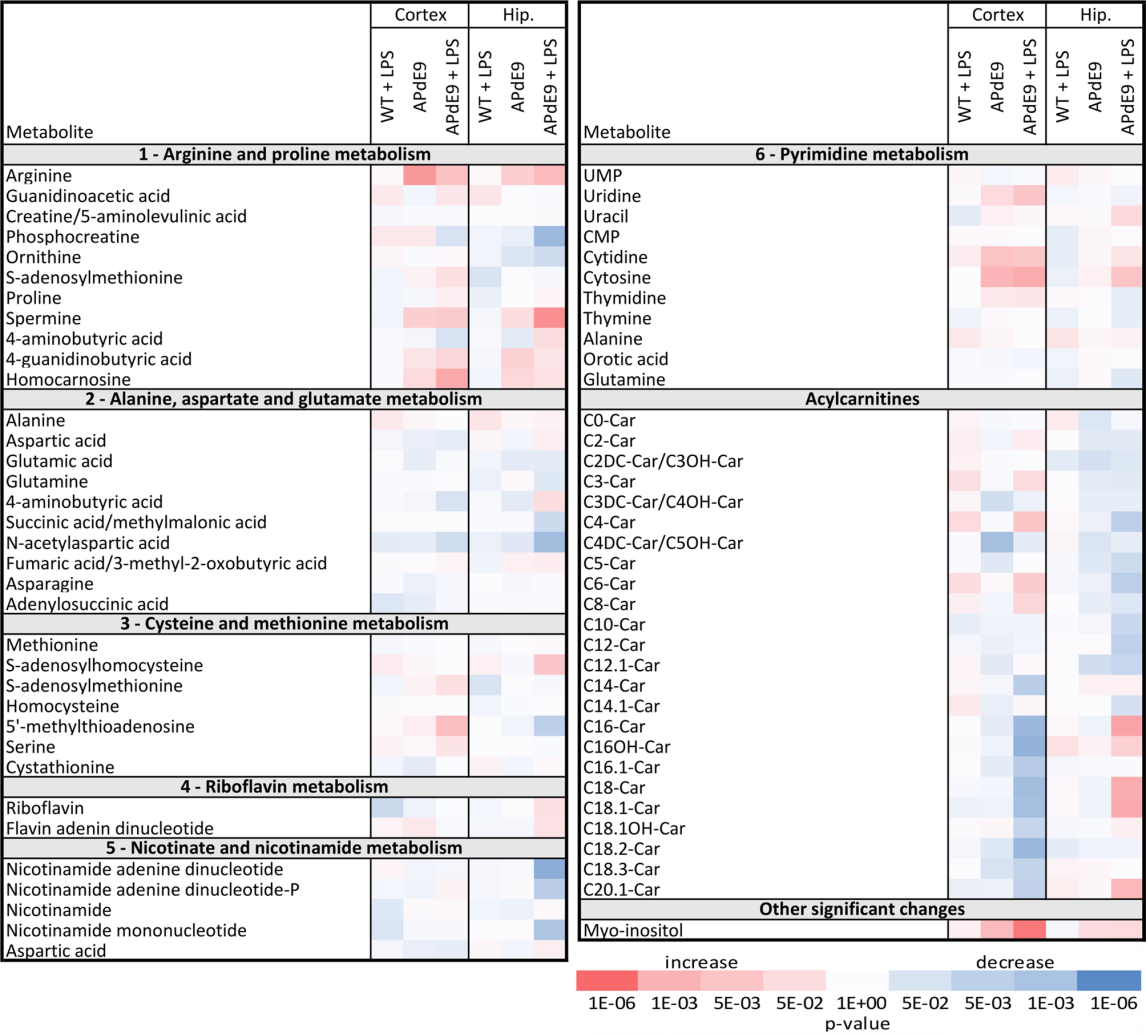
The heatmap of statistically significant and successfully annotated compounds represents the changes in metabolite levels in cortex and hippocampus of WT plus LPS (n = 11), APdE9 mice (n = 11), and APdE9 plus LPS (n = 9) groups versus WT control (n = 11). Heatmap of *p* values without correction, − log10 scaled: red—increased metabolites (fold-change > 0), blue—decreased metabolites (fold-change < 0). The statistical significance on the level of 0.05 after Bonferroni correction was calculated as a *p* value < 3.23 × 10^−4^. Details of the metabolites are provided in Supplementary Table S1 online.

To gain an in-depth insight into the individual metabolic pathways that were affected in the cortex and hippocampus, a *p* value based heatmap was constructed (Fig. 3, Supplementary Fig. S3). A gradual increase of statistical significance demonstrated as a decrease in the *p* value (Fig. 3, Supplementary Fig. S3, Supplementary Table S1 online) was observed for various metabolites in the APdE9 plus LPS mice compared to the APdE9 mice. According to the biochemical pathway analysis, LPS administration did not cause any significant changes in metabolite levels in the cortex and hippocampus of WT mice. The significant increase in arginine and *N*-formylglycinamide ribonucleotide was observed in the cortices of the APdE9 mice. The LPS treatment in the the APdE9 mice significantly increased the cortical levels of lysine, myo-inositol and hippocampal levels of spermine. Moreover, the reduction the hippocampal phosphocreatine levels and several metabolites involved in nicotinamide metabolism were observed in the APdE9 plus LPS mice. (Fig. 3, Supplementary Table S1 online). Interestingly, there was a decreasing trend in the levels of various short- and medium-chain acylcarnitines in the hippocampus of the APdE9 mice and APdE9 plus LPS mice compared to the WT control animals (Fig. 3, Supplementary Table S1 online). Similarly, long-chain acylcarnitines demonstrated a decreasing trend in the cortex of these two models as compared to the WT control. Statistically significant changes were observed for acylcarnitines (C16, C18:2, C16OH) in the APdE9 plus LPS mice compared to WT control group (Fig. 3, Supplementary Table S1 online).

### Untargeted lipidomic analysis of the brain tissue (cortex and hippocampus)

**Figure 4:**
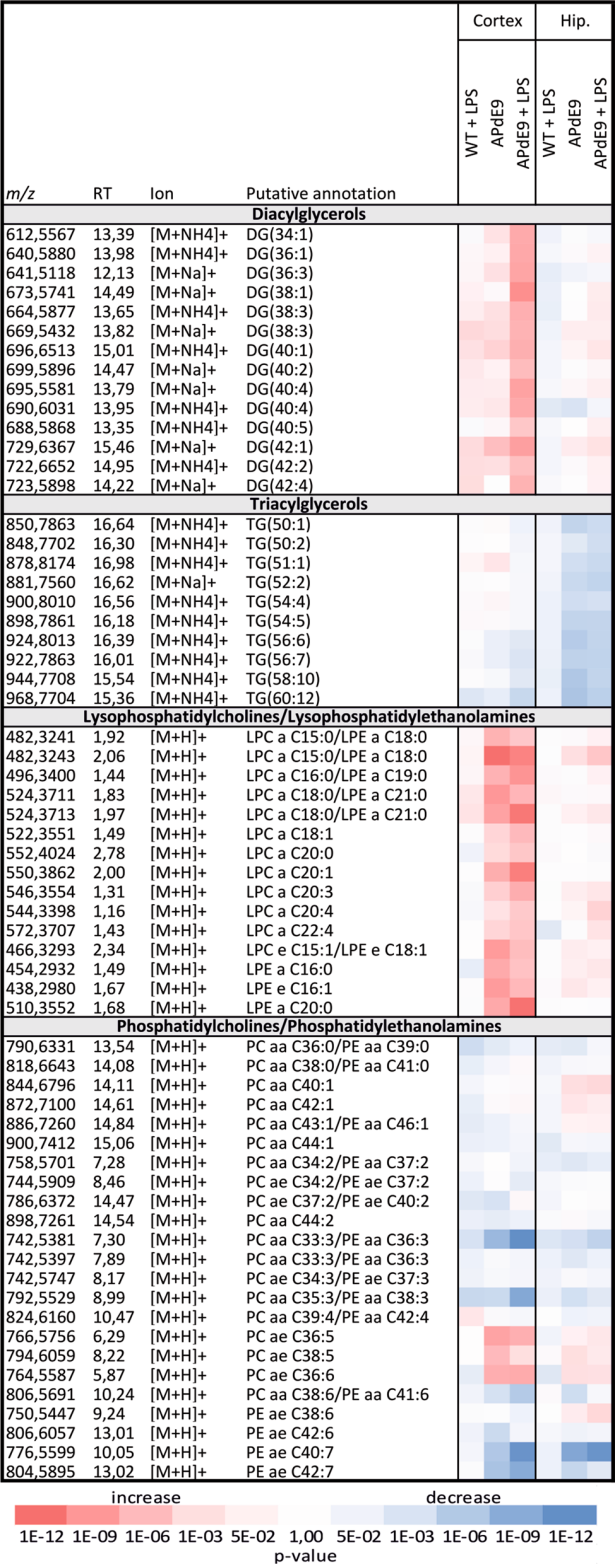
The heatmap of statistically significant and successfully annotated compounds represents the changes in the most affected lipid levels in the cortex and hippocampus of WT plus LPS (n = 11), APdE9 mice (n = 11), and APdE9 plus LPS (n = 9) groups versus WT control (n = 11). Heatmap of *p* values without correction − log10 scaled, red—increased lipids (fold-change > 0), blue—decreased lipids (fold-change < 0). The statistical significance after Bonferroni correction was considered as a *p* value < 3.48 × 10^−5^. Details of the metabolites are provided in Supplementary Table S2 online.

A *p* value based heatmap of the most affected lipids in the brain tissue was constructed (Fig. 4). The following significantly altered lipid classes were selected to display the general changes in the studied murine models compared to WT controls: phosphatidylcholines (PCs), phosphatidylethanolamines (PEs), lysophosphatidylcholines (LPCs), lysophosphatidylethanolamines (LPEs), diacylglycerols (DGs), triacylglycerols (TGs). The administration of LPS had no significant effect on cortical and hippocampal lipid metabolism in WT mice. Various DGs were significantly increased in the LPS-treated APdE9 mice (DG36:1, DG36:3, DG38:3, DG40:5, DG38:1, DG40:1, DG40:4, DG40:2, DG42:2, DG42:4, DG42:1) and the APdE9 mice (DG40:1, DG42:1) as compared to WT controls (Fig. 4, Supplementary Table S2 online). In contrast, the brain tissue TGs were not significantly altered in all three investigated models compared to WT controls. The hippocampal TGs showed decreasing trends in the APdE9 and APdE9 plus LPS groups as compared to WT controls. The LPCs/LPEs showed increasing trends in the APdE9 and APdE9 plus LPS mice compared to WT control, with significant increase in the level of various LPCs/LPEs (LPC e C15:1/LPE e C18:1, LPC a C15:0/LPE a C18:0, LPC a C15:0/LPE a C18:0, LPC a C16:0/LPE a C19:0, LPC a C18:1, LPC a C18:0/LPE a C21:0, LPC a C20:0, LPC a C20:1, LPC a C20:3, LPE a C20:0, LPE e C16:1, LPE a C16:0) in the cortex of both models. Various significant changes in PCs/PEs were found in the cortex and hippocampus of the APdE9 and APdE9 plus LPS mice compared to WT controls: decreased cortical PC aa C33:3/PE aa C36:3 (both groups), increased hippocampal PE ae C38:6 and PC aa C40:1 (APdE9 plus LPS ), decreased PE ae C40:7 (cortex APdE9 plus LPS and hippocampus both), decreased PC aa C35:3/PE aa C38:3 and PE ae C42:7 (cortex APdE9 plus LPS), increased cortical PC ae C36:6 and PC ae C36:5 (both groups), increased cortical PC ae C38:5 (APdE9 mice) (Fig. 4, Supplementary Table S2 online).

### Targeted metabolomic and untargeted lipidomic analysis of the plasma

The targeted metabolomic approach with both unsupervised PCA (Fig. 1c) and pathway analysis (Fig. 2) did not reveal significant metabolic changes in the plasma of the WT plus LPS group, the APdE9 and APdE9 plus LPS mice compared to the WT control. Thus, the metabolic alterations observed in the brain tissue were not fully reflected in the plasma metabolite levels (Supplementary Fig. S3), suggesting different regulation between the plasma and brain tissue metabolite levels. The untargeted lipidomic analysis revealed significantly reduced levels of monoacylglycerols (MG16:0, MG18:0), DGs (DG34:0, DG36:0, DG38:0, DG38:1) and ceramides (Cer d18:0/13:0, Cer d18:0/15:0, Cer d18:0/17:0) in the WT plus LPS group, the APdE9 group and the APdE9 plus LPS mice compared to WT controls. The levels of various PCs/PEs were significantly elevated in the WT plus LPS group, the APdE9 group and the APdE9 plus LPS mice compared to WT controls (Supplementary Table S4 online).

### Cytokine analysis in brain and plasma

**Figure 5:**
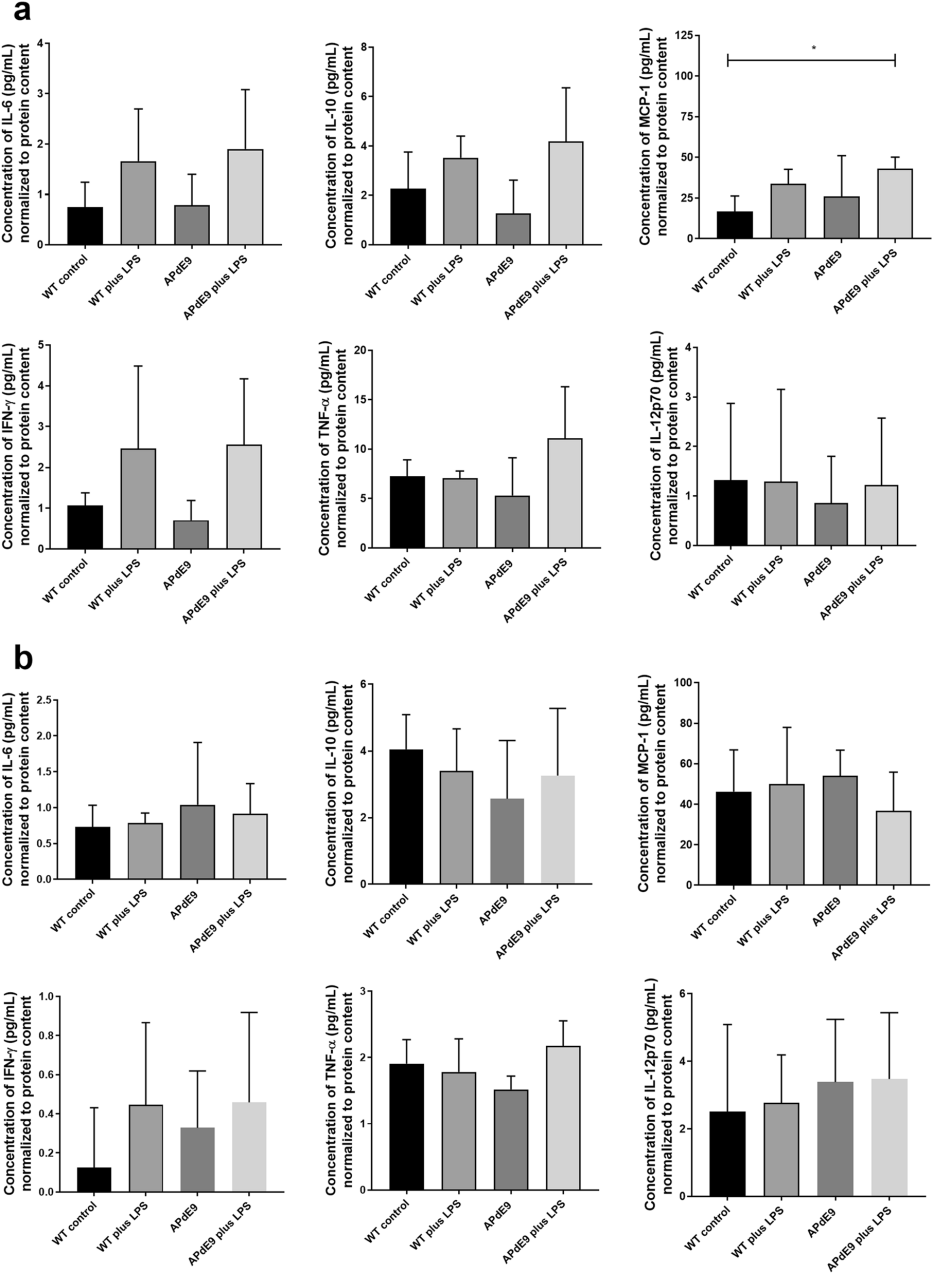
(**a**) The comparison of levels of interleukins (IL-6, IL-10, IL-12p70), interferon-γ (IFN-γ), monocyte chemoattractant protein-1 (MCP-1) and tumour necrosis factor α (TNF-α) in spleen of wild-type (WT) mice treated with LPS (n = 5), APdE9 mice with (n = 6) and without (n = 6) treatment with LPS versus WT control (n = 5). (**b**) The comparison of levels of IL-6, IL-10, MCP-1, IFN-γ, TNF-α and IL-12p70 in plasma of wild-type (WT) mice treated with LPS (n = 5), APdE9 mice with (n = 6) and without (n = 6) treatment with LPS versus WT control (n = 5). The data is presented as mean ± SD. Asterisks denote a statistically significant difference from the respective control (*p < 0.05, one-way ANOVA followed by Dunnett’s multiple comparisons test).

No differences in the production of the pro-inflammatory cytokines, i.e. interleukins (IL-6, IL-10, IL-12p70), interferon-γ (IFN-γ), and tumour necrosis factor α (TNF-α), were detected in the spleen and plasma of three mouse models compared to WT control group (Fig. 5a,b), with the exception of the elevated monocyte chemoattractant protein-1 (MCP-1) production in the APdE9 plus LPS mice compared WT mice (*p* < 0.05).

## Discussion

Systemic inflammatory conditions resulting from infections can be associated with increased risk of AD and accelerate the disease progression. Yet, the alterations of metabolic and lipid profiles due to the infection-driven systemic inflammation in AD have not been studied. To the best of our knowledge, this is the first study to reveal the brain and plasma metabolome and lipidome changes associated with systemic inflammation induced by administration of LPS in a transgenic AD mouse model, APdE9 mice. After comparing the metabolic and lipidomic profile in LPS-treated WT and APdE9 mice as well as saline-treated APdE9 mice to saline-treated WT controls, we revealed more significant alterations in cortical and hippocampal metabolome and lipidome in APdE9 mice treated with LPS compared to saline-treated APdE9 mice. In contrast, the impact of LPS administration on the cortical and hippocampal metabolome and lipidome in WT mice was negligible. These findings provide evidence that infection-induced systemic inflammation accelerate the changes in biochemical pathways in AD mice, but not in WT animals.

### Changes in metabolic pathways

The systemic administration of LPS increased the number of altered metabolic pathways in the brain tissue of the APdE9 mice. The biochemical pathways affected by LPS administration in the APdE9 mice, which have been previously reported to be altered in the brains of AD patients were cysteine and methionine metabolism^21^, arginine and proline metabolism^21^, nicotinate and nicotinamide metabolism^22^, alanine, aspartate, and glutamate metabolism^23^, glutathione metabolism^24^ and carbohydrate metabolism^25^, arachidonic acid metabolism^26^, fatty acid degradation^27^ and β-alanine metabolism^28^. The mentioned pathways were not altered in the brain tissue of the saline-treated APdE9 mice. These findings provide evidence that the changes in the mentioned metabolic pathways observed in AD patients may be triggered by systemic inflammation.

Moreover, a closer look at the brain tissue metabolic and lipid profile of the APdE9 mice after LPS administration revealed significant changes in the levels of several metabolites and lipids. In the saline-treated APdE9 mice, these changes were not significant but showed similar trend providing the evidence of accelerating effect of systemic inflammatory insult on brain metabolome and lipidome. For instance, similar to AD patients, significantly increased polyamine spermine levels in the hippocampus^29^ and cortical myo-inositol, a key molecule in cellular signal transduction and osmoregulation^30^, were observed in APdE9 mice after LPS administration compared to WT controls, but not in the saline-treated APdE9 mice. In addition, the levels of hippocampal NAD^+^ were significantly decreased after LPS treatment in the APdE9 mice compared to the WT controls. NAD^+^ is an essential metabolite for mitochondrial biogenesis and neuronal stress resistance acting as a co-factor for the DNA repair protein PARP1, for sirtuins (SIRT1 to SIRT7), and for the activity of the cyclic ADP ribose hydrolases CD38 and CD157. Hou et al. (2018) demonstrated that cortical NAD^+^ levels were decreased in an AD mouse model with introduced DNA repair deficiency (3xTgAD/Polβ + / −), and NAD + supplementation with nicotinamide riboside meaningfully normalised neuroinflammation, synaptic transmission, DNA damage, phosphorylated tau, improved learning, and memory functions. Our results support the vital role of NAD^+^ in AD pathogenesis and provide additional information about the possible role of systemic inflammation in NAD^+^ associated perturbations.

The hippocampal levels of phosphocreatine, a metabolite involved in the arginine cycle, were significantly decreased after LPS administration in the APdE9 mice while being reported to be elevated in AD patients compared to non-demented individuals^31^. Interestingly, LPS administration significantly increased the cortical levels of lysine in the APdE9 mice, whereas decreased levels of this amino acid were found in the brain of AD patients^32^. These results indicate that further studies are required to elucidate the impact of systemic inflammation on individual pathways involving the metabolites, which levels were altered in the present study.

LPS administration in the APdE9 mice had a significant impact on levels of several acylcarnitines (C16, C18:2, C16OH), while statistically insignificant trends were observed in the brain tissue of the saline-treated APdE9 mice as compared to WT controls. While the information about the changes in acylcarnitine brain tissue levels in AD is limited, our data indicate that further investigation of changes in acylcarnitines in AD would be of interest. Importantly, none of the above mentioned metabolic changes in the brain tissue were detected in the plasma of the APdE9 mice treated with LPS.

### Changes in lipidome

The LPS administration in the APdE9 mice resulted in greater number of significant changes in the brain lipids of the model compared to those observed in the saline-treated APdE9 mice. In the brain, lipids play an essential role in the structural integrity of neuronal membranes and regulation of neuronal activity and serve as precursors of bioactive lipid mediators^33^. Abnormal lipid metabolism in AD patients have been previously reported^34^. In the present study, the LPS administration significantly elevated levels of the brain cortical DGs in APdE9 mice (DG 36:1, DG36:3, DG38:3, DG40:5, DG38:1, DG40:1, DG40:4, DG40:2, DG42:2, DG42:4, DG42:1), while only DG 40:1 and DG42:1 were significantly increased in the cortex of the saline-treated APdE9 mice as compared to WT control animals. Similarly, augmented DGs’ levels in the frontal cortex of AD patients have been reported^35–37^. DGs act as structural lipids, signal transduction mediators, and precursors for glycerophospholipid. The tissue content of DGs is tightly regulated to maintain their diverse functions via several metabolic pathways^38^. Thus, Wood et al. (2015) suggested that elevated DGs in AD can be caused by glycerophospholipid degradation, which involves direct PE degradation to DGs driven by phospholipase C (PLC) and the deacylation of PE to LPE mediated by phospholipase A_2_ (PLA_2_) followed by the metabolism of LPE to DG^37^. The involvement of these pathways was supported by decreased PE levels observed in AD brains^35,36^ and plasma^39^. In the present study, various significant changes in the brain PE levels were found in the LPS-treated APdE9 mice. The cortical LPEs (LPE a C20:0, LPE e C16:1, LPE a C16:0) were significantly increased in both LPS-treated and saline-treated APdE9 mice compared to WT controls suggesting that systemic inflammation driven by LPS administration does not cause an effect on the brain LPE in APdE9 mice. There is a lack of information about the alterations in the LPE levels in AD brains. Based on the result of this study, we cannot provide conclusive evidence that the increase in DGs observed after LPS administration in APdE9 mice was due to PE degradation. Further studies are required to elucidate the underlying mechanism of these changes. Interestingly, elevated LPE levels were observed in the plasma of the LPS- and saline-treated APdE9 mice, which is in line with the previously reported increased plasma LPE 16:0 and 18:1 in AD patients^40^. Thus, if the alteration in LPE levels is confirmed in AD brains, these findings can open new opportunities to validate increased LPE levels in plasma as a biomarker for AD.

LPCs are generated from PCs by PLA_2_ and rapidly acylated with acetyl-CoA to maintain normal neural membrane composition. In the present study, the cortical levels of LPC and PC were significantly altered in both the LPS-treated and saline-treated APdE9 mice compared to WT controls in a similar manner. From these results, we draw the preliminary conclusion that LPS does not affect the formation of LPCs from PCs. Interestingly, the cortical LPC levels in the APdE9 mice were significantly elevated compared to WT controls, supporting the previously proposed LPC association with AD^41^. Similarly to our findings, increased levels of LPCs (LPC a C18:1 and LPC a C18:2) in the frontal cortex of post-mortem AD brains have been reported^42^.

### Region-specific changes in brain metabolome and lipidome

From a global perspective, the present study revealed some discrepancies in the metabolite and lipid changes between the hippocampus and cortex in both LPS-treated and saline-treated APdE9 mice compared to WT control animals. Targeted metabolomic analysis revealed that the hippocampus was slightly more affected than the cortex of the saline-treated APdE9 mice compared to WT control animals (Fig. 3). Similarly, the administration of LPS in the APdE9 mice resulted in more affected pathways in the hippocampus than in cortex. For instance, LPS administration altered the cysteine and methionine metabolism only in the cortex of the APdE9 mice, while arginine biosynthesis, glutathione synthesis, and alanine, aspartate and glutamate metabolism were affected only in the hippocampus. These findings are in line with the recent studies on region-specific proteomic and metabolic perturbations in the human AD brain, which showed that the hippocampus was more affected than the motor and sensory cortices^32,43^.

In contrast, in the present study, the cortex was more prone to lipid metabolism changes than the hippocampus in both the LPS- and saline-treated APdE9 mice compared to WT control animals. Lipids, the content of which in the brain is highly abundant and characterised by specific composition in different brain regions, are implicated in AD pathogenesis via regulation of the trafficking and proteolytic activity of several proteins bound to the plasma membrane, i.e. APP, β-secretase 1, and PSENs^33,44,45^. Our findings highlight the importance of investigation of the brain region-specific biochemical perturbations in AD, which currently lack systemic knowledge.

### Impact of peripheral inflammation on metabolic and lipidomic changes

Peripheral inflammation is manifested by elevated blood levels of circulating pro-inflammatory cytokines and chemokines. The increased levels of peripheral cytokines, such as TNF-α, IL-1β, IL-6, IL-12, IL-18 and transforming growth factor (TGF-β) were found in AD patients compared to healthy individuals^46^. The peripheral pro-inflammatory cytokines and chemokines stimulate a pro-inflammatory environment in the central nervous system by several routes, including signalling via endothelial cells and circumventricular organs and the vagus nerve stimulation^10^. Through each of these routes, systemic inflammation is thought to induce reactive microglia and astrocytic phenotypes which can promote tau hyperphosphorylation, oligomerisation of Aβ, complement activation, and the biochemical perturbations^10^. The LPS treatment paradigm used in the present study has previously induced inflammation in the brain of aged 3xTg-AD mice as demonstrated by more CD45 + infiltrated macrophages and microglia and by increased levels of pro-inflammatory cytokines with no effect on amyloid plaque load^47^. The interpretation of the observed changes in metabolome and lipidome and their association to systemic inflammation was made based on the assumption, that the similar effects were achieved after the LPS treatment in APdE9 mice. Moreover, NOR test demonstrated decreased recognition memory in LPS-treated WT and APdE9 mice compared to WT controls, which is in accordance with the previous reports on the changes in recognition memory due to the infection-induced inflammation^48,49^. However, in the present study, we did not observe the significant changes in the production of the pro-inflammatory cytokines in the plasma and spleen of the investigated models compared to WT animals. The only inducing effect on the production of MCP-1 in the spleen, but not in plasma was observed in APdE9 mice treated with LPS as compared to WT control mice. Seemann et al. (2017) demonstrated that systemic LPS administration causes a transient increase in pro-inflammatory cytokines in mice, which explains the absence of peripheral cytokine changes after a five-week wash-out before the sample collection^50^. Therefore, one can assume that the LPS-induced changes in the metabolic and lipid profile in the brain of the APdE9 mice result from the chronic brain-specific pathophysiological processes, which are triggered but not maintained by systemic inflammation. On the other hand, there is evidence that repeated administrations of LPS induce a state of LPS-tolerance characterized by a reprogramming of TLR 4-bearing cells and as a result switching from the pro- to anti-inflammatory state^51–53^. In this case, the findings of the present study will demonstrate that the manifestation of LPS-tolerance in WT and transgenic AD mice is different. Thus, the future studies should elucidate the particular mechanisms (e.g. tolerance, neuroinflammation) by which LPS-induced systemic inflammation causes the metabolic and lipidomic changes in the brains of APdE9 mice.

### Limitations and future perspectives

This study aimed to monitor general changes in the metabolome and lipidome in mouse models in order to give first insight about the altered biochemical pathways that should be more thoroughly investigated in the future. Here, we reported the changes in the brain cortical and hippocampal tissue and, therefore, the observed alterations cannot be attributed to the specific brain parenchymal cells. However, it should be noted that neuroinflammation and AD pathology causes various changes in individual brain parenchymal cells. For example, Aβ triggers acute microglial inflammation accompanied by metabolic reprogramming from oxidative phosphorylation to glycolysis in order to support the synthesis and release of pro-inflammatory mediators^54,55^. The activated microglia consequentially reaches a chronic tolerant state as a result of comprehensive defects in energy metabolisms and subsequently diminished immune responses, such as cytokine production and phagocytosis^55^. Moreover, the major pool of brain lipids is found in the myelin sheath, which has been shown to be disrupted due to Aβ deposition resulting in degradation of white matter^56^. Therefore, the future studies have to shed light on the cell-specific alterations in molecular mechanisms underlying the metabolic and lipidomic changes observed in the present study.

Importantly, the LPS regimen has been selected based on the previous reports, as it demonstrated increased levels of pro-inflammatory cytokines in the brain of transgenic AD mice^47^. Therefore, we assumed to see similar changes in our study. However, due to limited availability of the cortical and hippocampal samples the cytokine analysis in the present study has not been performed. In addition, the effect of chronic LPS treatment on Aβ deposition in APdE9 mice was not investigated in this study. Therefore, future studies should focus on investigation of the causative factors of the observed changes in metabolome and lipidome of APdE9 mice.

Moreover, despite the technological progress over the past decade, the field of metabolomics is still facing many challenges when it comes to pathway mapping and metabolite identification/quantification. Many of the metabolites are below the limit of detection/quantification of current analytical protocols and/or are not present in spectrum libraries, making their analyses challenging. Therefore, future work should focus on investigating specific metabolites and pathways.

## Conclusion

In conclusion, the present study is the first extensive investigation that characterises the changes in the plasma and brain metabolome and lipidome triggered by LPS-induced systemic inflammation in transgenic APdE9 mice. The findings of this study demonstrated that systemic inflammation caused additional significant biochemical perturbations in the transgenic AD mice, but not in wild-type animals. Similar changes have been previously reported in AD patients. These results suggest that infection-induced systemic inflammation may accelerate the changes in biochemical pathways in AD patients, but not in healthy individuals. This information benefits the understanding of the impact of infection-driven systemic inflammation on AD progression.

## Methods

### Materials

Tris–HCl, sucrose, EDTA, EGTA, methanol, tert-butyl methyl ether, propan-2-ol, acetonitrile, dichloromethane, LPS (#L2880), tribromoethanol (#75-80-9, Avertin) were purchased from Sigma-Aldrich (St. Louis, MO, USA). Pierce BCA Protein Assay Kit was purchased form the Thermo Fisher Scientific, Thermo Scientific™.

### Study design and animals

The animal experiments complied with the ARRIVE guidelines and were conducted according to EU Directive 2010/63/EU for animal experiments. The procedures involving the animal use were approved by the Finnish National Animal Experimental Board (ESAVI-2015-000744). The inclusion/exclusion criteria were based on the health state of animals. The animals that were healthy and showed no sign of illness as evaluated by the body weight and visual observations were used in the analysis. None of the animals died during the study. Because of the higher prevalence of AD and risk for women compared to men^57^, female mice were used. With respect to the required power of the study set to 0.8 and alpha to 0.05, the effect size of 1.1 and higher was calculated as statistically significant for sample size (n = 9 per group) in the study. The sample size was set as a compromise between the complexity of the experiment, the use of animal models in the study and the general recommendation for metabolomic studies.

The animals belonged to one of four study groups: the WT mice with LPS administration (WT plus LPS group), APdE9 mice (APdE9 group, RRID: MGI:5701399)^14^ and APdE9 mice with LPS treatment (APdE9 plus LPS group), all of which were compared to age-matched WT mice (WT control group). The APdE9 transgenic mice used were generated by co-injection of chimeric mouse/human APP695 harbouring the Swedish mutation and human PS1-dE9 vectors, both controlled by their own mouse prion protein promoter element^14^. The double transgenic mice were backcrossed to C57BL/6 J strain to create APdE9 transgenic (APP/PS1) mice in C57BL/6 J background. Wild-type siblings were used as controls. The mice age was 16–17 months old, as we previously showed that at this age the APdE9 mice is characterized by increased formation of Aβ plagues and learning and memory deficits^8,20,56^. The LPS was chronically administered according to previously published protocol with small modifications^47,58^. The chronic LPS treatment used in the study have previously induced inflammation and impaired tau pathological characteristics leading to diminishing in spatial memory in aged 3xTg-AD mice^47^. Briefly, the mice in the WT plus LPS (n = 11) and APdE9 plus LPS (n = 9) groups were administered 500 µg/kg i.p. LPS twice a week for four weeks followed by a two-week wash-out period. After that, the animals were administered again 500 µg/kg i.p. LPS twice a week for four weeks followed by five weeks of wash-out with subsequent decapitation. The wash-out time was used to ensure that the observed changes in the lipidome and metabolome are chronic and maintained after the LPS dosing is ceased. The mice in the WT control (n = 11) and APdE9 (n = 10) groups were injected 0.9% saline solution i.p. instead of LPS according to the same regimen as in the WT plus LPS and APdE9 plus LPS groups. The behavioural tests such as Novel Object Recognition (NOR) test and Open Field Spontaneous Movement Activity test using TruScan were performed in order to characterize the mouse models in terms of recognition memory and motor activity (Supplementary Information 1).

All the animals were housed under standard laboratory conditions as follows: four to six animals per cage, 12–12 h light–dark day cycle, food (Lactamin R36; Lactamin AB, Södertälje, Sweden), and water consumption ad libitum, 60% relative humidity. On the decapitation day, the mice were anaesthetised by tribromoethanol, a terminal anaesthetic providing fast and deep surgical analgesia. The use of tribromoethanol was approved by the Finnish National Animal Experimental Board (ESAVI-2015–000,744). After anaesthesia the blood was collected by cardiac puncture into tubes containing citrate as an anticoagulant. All procedures were conducted during the daytime. The plasma was separated by centrifugation at 1500 × *g* for 6 min, after which the plasma layer was centrifuged again at 12,000 × *g* to remove the platelets. The plasma was stored at − 80 °C until the analysis. The mouse brains were perfused using 3-min transcardial perfusion with heparinised saline (2500 IU/L). After removal of the meninges, the brain cortex, hippocampus, and spleen were separated, snap-frozen in liquid nitrogen, and stored at − 80 °C until the analysis.

### Plasma sample preparation

#### Metabolite extraction from plasma samples for targeted metabolomics

The sample preparation of the plasma for targeted metabolomics was performed according to a previously described protocol^59^. Briefly, 30 μL of plasma samples were centrifuged at 14,000 × *g* for 10 min at 4 °C. The collected 20 μL of supernatants were mixed with 80 μL of pre-chilled methanol to make a final 80% (v/v) methanol solution. The samples were gently shacked and incubated for 6 h at − 80 °C, followed by centrifugation at 14,000 × *g* for 10 min at 4 °C. The supernatant (80 μL) was subsequently freeze-dried. Extract blanks were prepared by the same method, using water instead of plasma. The samples were stored at − 80 °C until the analysis.

#### Lipid-focused extraction from plasma samples for untargeted lipidomics

Lipids (non-polar phase) from the plasma samples were extracted using a biphasic extraction protocol adapted from Sarafian et al.^60^. The plasma samples (25 μL) were mixed with a mixture of methanol/tert-butyl methyl ether/water (187.5 μL; 1:5:1.5; vol/vol/vol) and vortexed for 60 s. The samples were left at room temperature for 10 min and incubated at − 20 °C overnight. Subsequently, the samples were centrifuged at 14,000 × *g* for 20 min at 4 °C, and the organic phase (120 μL) was collected and freeze-dried (FreeZone, Labconco) and stored at − 80 °C until the analysis. Extract blanks were prepared by the same method using water instead of plasma.

### Tissue sample preparation

The mouse cortex and hippocampus sample preparation was performed according to the previously described procedure^61^.

#### Tissue sample preparation for targeted metabolomics (aqueous extraction)

For aqueous extraction, frozen cortex and hippocampus samples (26.2 ± 4.26 mg) were transferred into bead beating tubes (Sample Tubes RB, QIAGEN) preloaded with one 5-mm steel bead (QIAGEN). Subsequently, a 50% (vol/vol) aqueous solution of pre-chilled methanol was added, with the adjusted volume based on the tissue weight (100 μL of solvent per 28 mg of tissue). After the samples had been frozen on dry ice, the lysis of the tissue and extraction of metabolites was performed using a bead beater (TissueLyser II, QIAGEN). The vibrating of the bead beater was 30 times/s for 40 s with 2 plus 2 cycles. The samples were centrifuged at 13,000 × *g* for 20 min at 4 °C. Aliquots of 100 μL of supernatant (28 mg of sample/100 μL of aliquot) were put into Eppendorf tubes and freeze-dried. The samples were stored at − 80 °C until the analysis.

#### Tissue sample preparation for untargeted lipidomics (organic extraction)

For organic extraction, a solution of pre-chilled dichloromethane/methanol (3:1) was added to the residual pellet obtained after aqueous extraction. The solution volume was adjusted to the sample weight (28 mg of tissue/100 μL of solvent). After freezing on dry ice, the samples were transferred into the bead beater (at 30 times/s, 40 s/cycle, 2 cycles) followed by centrifuging at 13,000 × *g* for 20 min at 4 °C. The aliquots of organic phase supernatant (28 mg of tissue/100 μL of aliquot) were transferred into glass vials. Subsequently, the samples were left in an extractor hood overnight for evaporation at room temperature. The samples were stored at − 80 °C until the analysis.

### Sample resuspension for targeted metabolomics and untargeted lipidomics

For the targeted metabolomic analysis, the samples were resuspended in 100 μL of methanol/water (4:1; vol/vol). Following the resuspension, the samples were vortexed (30 s). A pooled quality control (QC) sample was prepared by combining 10-μL aliquots from the resuspended extracts and thoroughly vortexed (5 min). All the samples were centrifuged (at 20,000 × *g* for 10 min at 4 °C) and loaded into high-performance liquid chromatography (HPLC) vials.

For the untargeted lipidomic analysis, the samples were prepared by precisely the same procedure as the samples for the targeted analysis using 120 μL of propan-2-ol/acetonitrile/water (2:1:1; vol/vol/vol) instead of 100 μL of a methanol/water mixture.

### LC/MS targeted metabolomics and untargeted lipidomics methods

The analytical method for targeted metabolomics was adopted from Yuan et al.^59^. The experiments were performed by UHPLC-MS/MS using a Dionex UltiMate 3000 Rapid Separation LC system (Thermo Fisher Scientific, MA, USA) coupled with an electrospray Triple Quad 6500 (SCIEX, Framingham, MA, USA). The metabolites were separated on a Phenomenex Luna NH2, 2.1 × 150 mm, 3 μm, column (Phenomenex, UK). Mobile phase A consisted of 20 mM ammonium acetate (pH 9.75) in water, while mobile phase B consisted of pure acetonitrile. The flow rate was set to 0.30 mL/min with the following gradient: t = 0.0, 95% B; t = 15.0, 30% B; t = 17.0, 5% B; t = 23.0, 5% B; t = 23.1, 95% B; t = 28.0 min 95% B. The column temperature was set to 35 °C, and the injection volume was 2 μL. The MS parameters were as follows: the ion spray voltage was + 5500 V and − 4500 V; curtain gas 40 psi; both ion source gases were 40 psi; ion source temperature 400 °C. The Analyst software (SCIEX) was used to control the LC/MS instrument.

The untargeted lipidomic study was performed by applying the ultra-high performance liquid chromatography-mass spectrometry (UHPLC-MS) method using a Dionex UltiMate 3000 Rapid Separation LC system (Thermo Fisher Scientific, MA, USA) coupled with an electrospray Orbitrap Elite mass spectrometer (Thermo Fisher Scientific, MA, USA). Lipid profiles were separated on an Acquity UPLC BEH C18 2.1 × 100 mm, 1.7 μm, column (Waters Corp, USA) using a modified previously published method (Vorkas et al.^61^). Mobile phase A consisted of 10 mM ammonium formate and 0.1% formic acid in 60% acetonitrile/water, and mobile phase B consisted of 10 mM ammonium formate and 0.1% formic acid in 90% propan-2-ol/water. The flow rate was set to 0.40 mL/min with the following gradient: t = 0.0, 40% B; t = 2, 43% B, t = 2.1, 50% B; t = 12.0, 54% B; t = 12.1, 70% B; t = 18.0, 99% B; t = 18.1, 40% B; t = 20.0, 40% B. The column temperature was set to 55 °C, and the injection volume was 2 μL. The data was acquired in positive ionisation mode within the mass range of 100–1600 m/z at a resolution of 60,000 FWHM. The ion source parameters were set as follows: the sheath gas was 40 arbitrary units; the aux gas was 15 arbitrary units; the spray voltage was 3 kV; the capillary temperature was 300 °C; the source heater temperature was 350 °C. Selected features were subjected to MS/MS analysis using different collision energies. A Thermo Tune Plus 2.7.0.1103 SP1 was used as the instrument control software for the Orbitrap Elite, and the data was acquired in centroid mode.

The QC samples were analysed as the first ten injections and then every sixth injection with two QC samples at the end of the analytical batch. Two blank samples were analysed, the first as the sixth injection and then at the end of each batch.

### Cytokine analysis

The cytokine analysis of the mouse spleen and plasma samples was performed to elucidate whether the changes in the metabolome and lipidome in the brain are driven by central or systemic inflammation. The frozen spleen tissue samples were weighed. Tissue Homogenising Buffer (THB) was added in the proportion of 8 μL/mg spleen tissue. The THB contained diethyl pyrocarbonate (DEPC) water, 20 mM Tris–HCl (pH 7.5), 250 mM sucrose, 5 mM EDTA, 10 mM EGTA, and a protease inhibitor cocktail (1:200). A homogenising bead was added to each tube and the tissue was homogenised with a bead beater (TissueLyser II, QIAGEN) at 30 Hz for 2 min at 4 °C. The samples were centrifuged at full speed (14,000 × *g*) at 4 °C for 10 min, and the supernatant was transferred to a new tube and stored at − 80 °C until the analysis. The protein analysis was performed with a Pierce BCA Protein Assay Kit, and the results of the Cytometric Bead Array (CBA) were normalised to the total protein concentrations of the samples.

The cytokine analysis of the mouse spleen and plasma samples was performed with a BD Cytometric Bead Array mouse inflammation kit (BD Biosciences San Diego, CA, USA) according to the manufacturer’s instructions. The levels of IL-6, IL-10, MCP-1, IFN-γ, TNF-α, and IL-12p70 in the plasma and spleen samples were measured. The samples were run with CytoFLEX S, and the results were analysed with the FCAP Array 2.0.0 software (Soft Flow Hungary Ltd, Pecs, Hungary). 

### Data analysis and statistics

Peak integration of the targeted metabolomics data was performed with MultiQuant 3.0 (SCIEX). The raw data files from untargeted lipidomics analysis were pre-processed by vendor software Compound Discoverer 3.0 (Thermo Fisher Scientific), including RT alignment, peak-picking, adduct annotation, and blank subtraction. In-source fragment removal was done by R-script CROP^62^. The data handling and statistical evaluation were performed on the basis of the R-package Metabol^63^, using R Software^64^. Quality control-based LOESS regression was applied^65^. On the basis of the coefficients of variation (CVs) calculated from the QC samples, metabolites/features with a CV higher than 30% were excluded from further data processing. The data were analysed as compositional using centred log-ratio (clr) coefficients and mean centring^66^. This approach combines logarithm transformation to normal distribution and scaling of the data based on geometric means. Both unsupervised principal component analysis (PCA) and supervised multivariate statistical approaches (partial least squares discriminant analysis, PLS-DA) were applied, as well as univariate statistical methods (*p* value based heatmaps, box plots). The box plots can be provided on request, as the file size exceeds the limits. The *p* value was calculated by means of a *t*-test, and Bonferroni correction was applied (α = 0.05/number of metabolites or features). The presence of outliers was evaluated by interquartile range outlier detection method and visual inspection of the results from multivariate statistics. The most altered biochemical pathways and their comparison for all the groups under study were determined using the MetaboAnalyst 4.0 Pathway Analysis module, which is based on the KEGG metabolic pathways database (140 metabolites from the Mus musculus library were used in this study) and combines the results from pathway enrichment analysis (represented by the x-axis) with pathway topology analysis (represented by the y-axis). In order to identify and directly compare the most relevant pathways, the − log(*p* value) threshold was set to 3.0, and the pathway impact value threshold was set to 0.3 (highlighted by the red rectangle in the graphs). The Global Test and Relative-betweenness Centrality algorithms were applied. For the purposes of the article, only statistically significant features based on a Metlin database match (mass tolerance < 1 ppm) and appropriate retention time behaviour were annotated. Some of the discriminating lipids, i.e. LPC/LPE and PC/PE, were not differentiated due to the same molecular weight and retention time behaviour.

For the statistical analysis of the differences in plasma and spleen cytokine levels (IL-6, IL-10, MCP-1, IFN-γ, TNF-α, and IL-12p70) between the groups, i.e. the WT plus LPS, APdE9, APdE9 plus LPS versus WT control, the one-way ANOVA followed by Dunnett's multiple comparisons test was used (GraphPad Prism 6, La Jolla, CA). Statistical significance was defined as *p* < 0.05.

## Supplementary Information

Supplementary Information 1.

Supplementary Information 2.

Supplementary Information 3.

## Data Availability

All the data generated or analysed during the study are included in this published article and its supplementary information files.
