# Supplementary material for: Metabolomic and lipidomic changes triggered by lipopolysaccharide-induced systemic inflammation in transgenic APdE9 mice
Source: Sci Rep. 2021 Jun 22;11:13076. doi: 10.1038/s41598-021-92602-4 (PMC8219693; doi:10.1038/s41598-021-92602-4)
Supplement: Supplementary file 1 — Supplementary Information 1. [file 41598_2021_92602_MOESM1_ESM.pdf]

Metabolomic and lipidomic changes triggered by lipopolysaccharide-induced systemic inflammation in transgenic APdE9 mice

Elena Puris[1,2†], Štěpán Kouřil[3,4†], Lukáš Najdekr[3], Sanna Loppi[5,6], Paula Korhonen[5], Katja M. Kanninen[5], Tarja Malm[5], Jari Koistinaho[5,7], David Friedecký[3,4], Mikko Gynther[1]*

[1]School of Pharmacy, University of Eastern Finland, P.O. Box 1627, 70211 Kuopio, Finland

[2]Institute of Pharmacy and Molecular Biotechnology, Ruprecht-Karls-University, Im Neuenheimer Feld 329, 69120 Heidelberg, Germany (present address for E.P.)

[3]Institute of Molecular and Translational Medicine, Palacký University Olomouc, Hněvotínská 5, 77900 Olomouc, Czech Republic

[4]Department of Clinical Biochemistry, University Hospital Olomouc, I.P. Pavlova 6, 77900 Olomouc, Czech Republic

[5]A.I. Virtanen Institute for Molecular Sciences, University of Eastern Finland, P.O. Box 1627, 70211 Kuopio, Finland

[6]Department of Immunobiology, University of Arizona, 1656 E Mabel Street, Tucson, Arizona 85724-5221, USA (present address for S.L.)

[7]Neuroscience Center, Helsinki Institute for Life Science, University of Helsinki, Haartmaninkatu 8, 00290 Helsinki, Finland (present address for J.K.)

[†]Elena Puris and Štěpán Kouřil should be considered joint first authors.

***corresponding author: Elena Puris, Institute of Pharmacy and Molecular Biotechnology, Ruprecht-Karls-University, Im Neuenheimer Feld 329, 69120 Heidelberg, Germany; phone: +(358)449789164; email: elena.puris@uni-heidelberg.de

Supplementary Figure S3. The heatmap of statistically significant and successfully annotated compounds represents the changes in metabolite levels in cortex, hippocampus and plasma of WT plus LPS (n = 11), APdE9 mice (n = 11), and APdE9 plus LPS (n = 9) groups vs. WT control (n = 11). Heatmap of *p*-values without correction, – log10 scaled: red – increased metabolites (fold-change > 0), blue – decreased metabolites (fold-change < 0). The statistical significance on the level of 0.05 after Bonferroni correction was calculated as a *p*-value < $3.23*10^{-4}$. Details of the metabolites are provided in Supplementary Table S1 and S3 online.

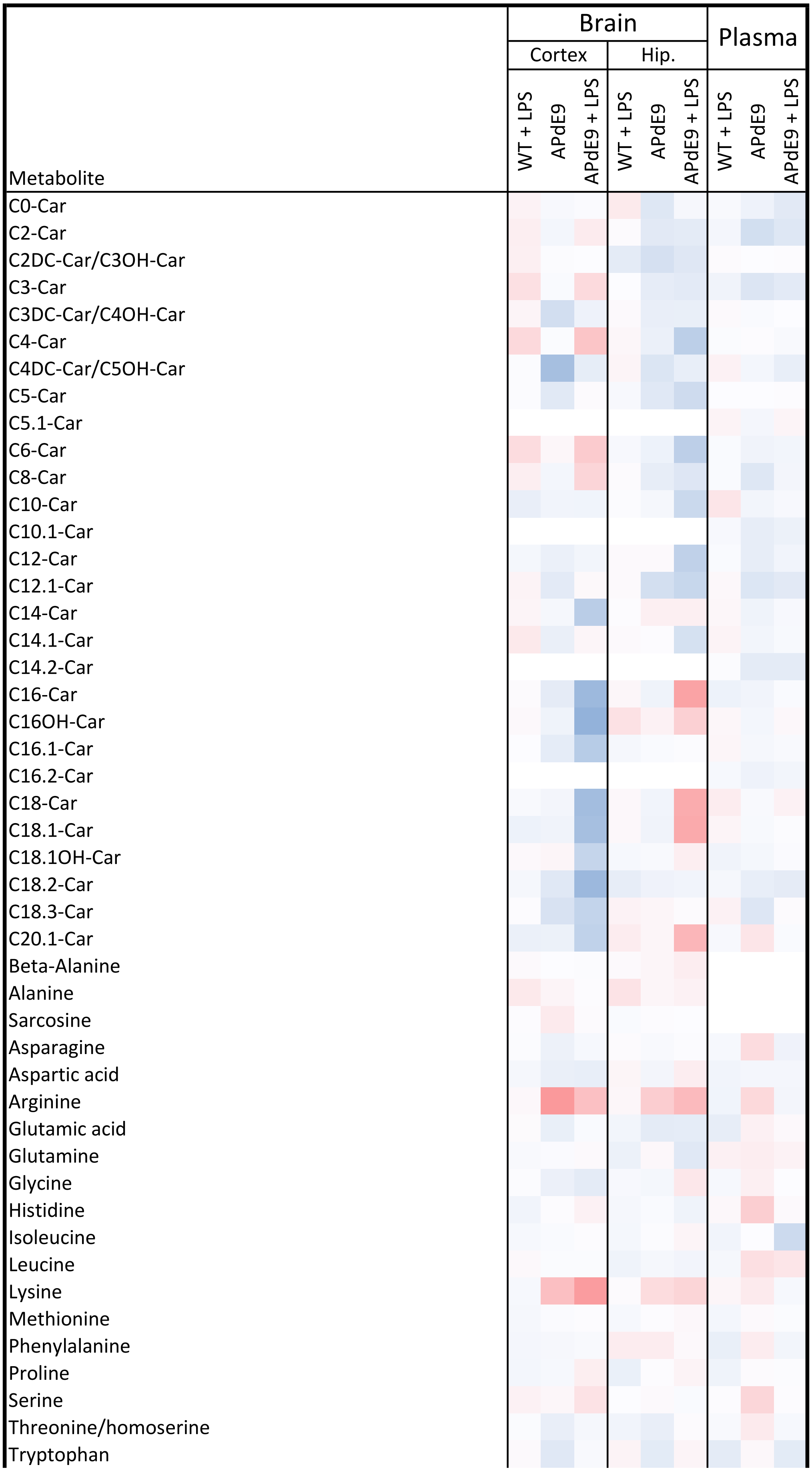
Brain
Plasma
Cortex
Hip.
WT + LPS
APdE9
APdE9 + LPS
WT + LPS
APdE9
APdE9 + LPS
WT + LPS
APdE9
APdE9 + LPS
Metabolite
C0-Car
C2-Car
C2DC-Car/C3OH-Car
C3-Car
C3DC-Car/C4OH-Car
C4-Car
C4DC-Car/C5OH-Car
C5-Car
C5.1-Car
C6-Car
C8-Car
C10-Car
C10.1-Car
C12-Car
C12.1-Car
C14-Car
C14.1-Car
C14.2-Car
C16-Car
C16OH-Car
C16.1-Car
C16.2-Car
C18-Car
C18.1-Car
C18.1OH-Car
C18.2-Car
C18.3-Car
C20.1-Car
Beta-Alanine
Alanine
Sarcosine
Asparagine
Aspartic acid
Arginine
Glutamic acid
Glutamine
Glycine
Histidine
Isoleucine
Leucine
Lysine
Methionine
Phenylalanine
Proline
Serine
Threonine/homoserine
Tryptophan

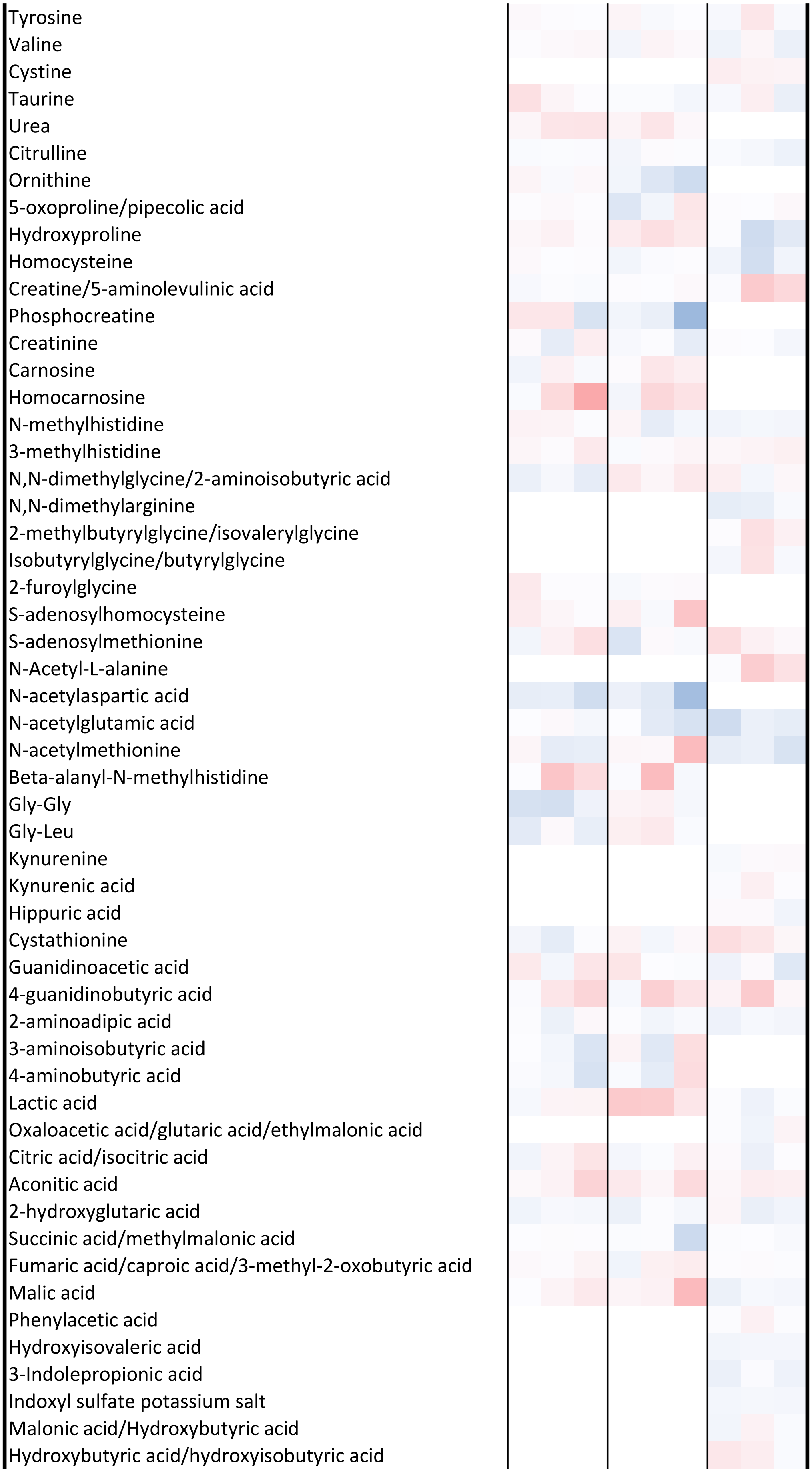
Tyrosine
Valine
Cystine
Taurine
Urea
Citrulline
Ornithine
5-oxoproline/pipecolic acid
Hydroxyproline
Homocysteine
Creatine/5-aminolevulinic acid
Phosphocreatine
Creatinine
Carnosine
Homocarnosine
N-methylhistidine
3-methylhistidine
N,N-dimethylglycine/2-aminoisobutyric acid
N,N-dimethylarginine
2-methylbutyrylglycine/isovalerylglycine
Isobutyrylglycine/butyrylglycine
2-furoylglycine
S-adenosylhomocysteine
S-adenosylmethionine
N-Acetyl-L-alanine
N-acetylaspartic acid
N-acetylglutamic acid
N-acetylmethionine
Beta-alanyl-N-methylhistidine
Gly-Gly
Gly-Leu
Kynurenine
Kynurenic acid
Hippuric acid
Cystathionine
Guanidinoacetic acid
4-guanidinobutyric acid
2-aminoadipic acid
3-aminoisobutyric acid
4-aminobutyric acid
Lactic acid
Oxaloacetic acid/glutaric acid/ethylmalonic acid
Citric acid/isocitric acid
Aconitic acid
2-hydroxyglutaric acid
Succinic acid/methylmalonic acid
Fumaric acid/caproic acid/3-methyl-2-oxobutyric acid
Malic acid
Phenylacetic acid
Hydroxyisovaleric acid
3-Indolepropionic acid
Indoxyl sulfate potassium salt
Malonic acid/Hydroxybutyric acid
Hydroxybutyric acid/hydroxyisobutyric acid

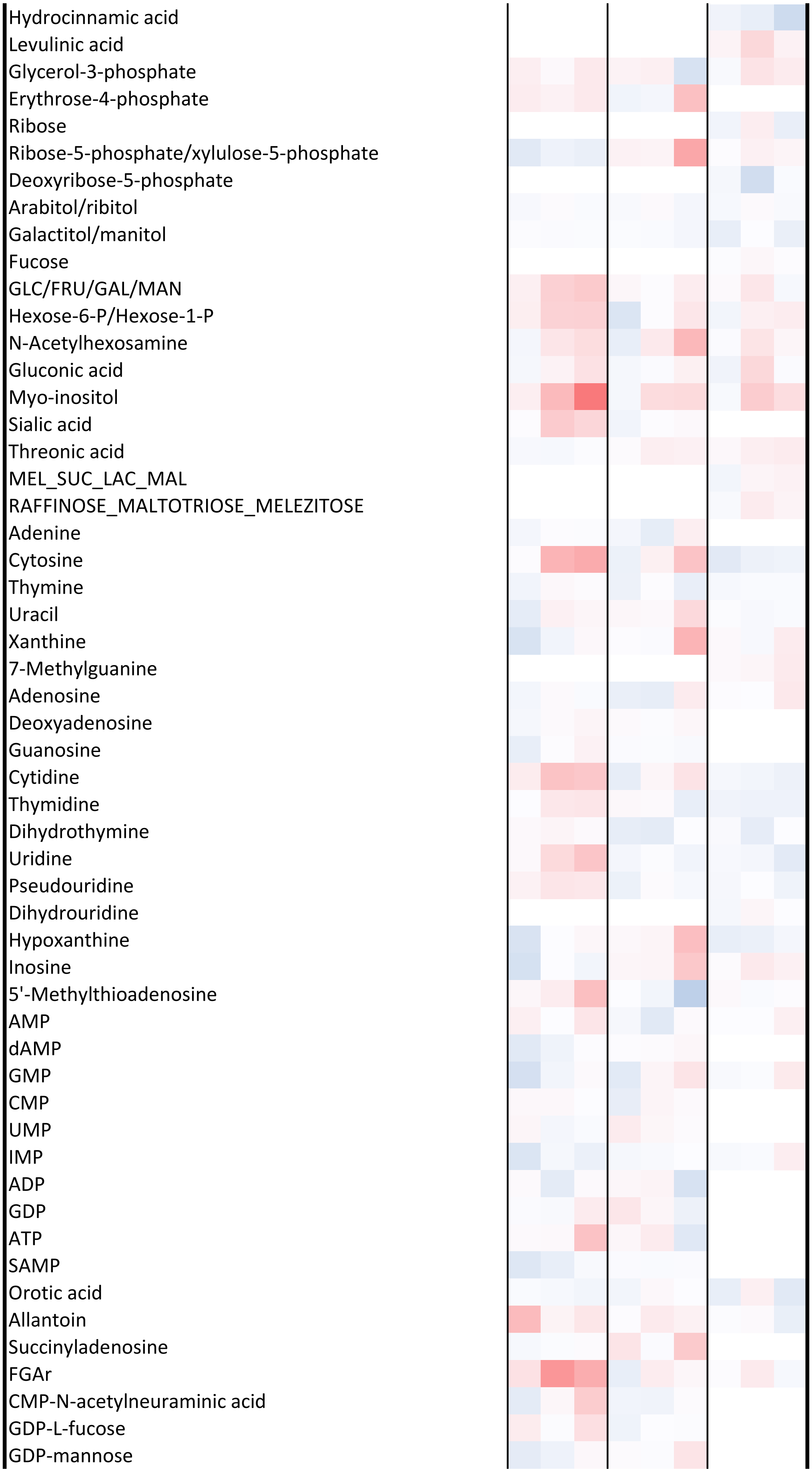
Hydrocinnamic acid
Levulinic acid
Glycerol-3-phosphate
Erythrose-4-phosphate
Ribose
Ribose-5-phosphate/xylulose-5-phosphate
Deoxyribose-5-phosphate
Arabitol/ribitol
Galactitol/manitol
Fucose
GLC/FRU/GAL/MAN
Hexose-6-P/Hexose-1-P
N-Acetylhexosamine
Gluconic acid
Myo-inositol
Sialic acid
Threonic acid
MEL_SUC_LAC_MAL
RAFFINOSE_MALTOTRIOSE_MELEZITOSE
Adenine
Cytosine
Thymine
Uracil
Xanthine
7-Methylguanine
Adenosine
Deoxyadenosine
Guanosine
Cytidine
Thymidine
Dihydrothymine
Uridine
Pseudouridine
Dihydrouridine
Hypoxanthine
Inosine
5'-Methylthioadenosine
AMP
dAMP
GMP
CMP
UMP
IMP
ADP
GDP
ATP
SAMP
Orotic acid
Allantoin
Succinyladenosine
FGAr
CMP-N-acetylneuraminic acid
GDP-L-fucose
GDP-mannose

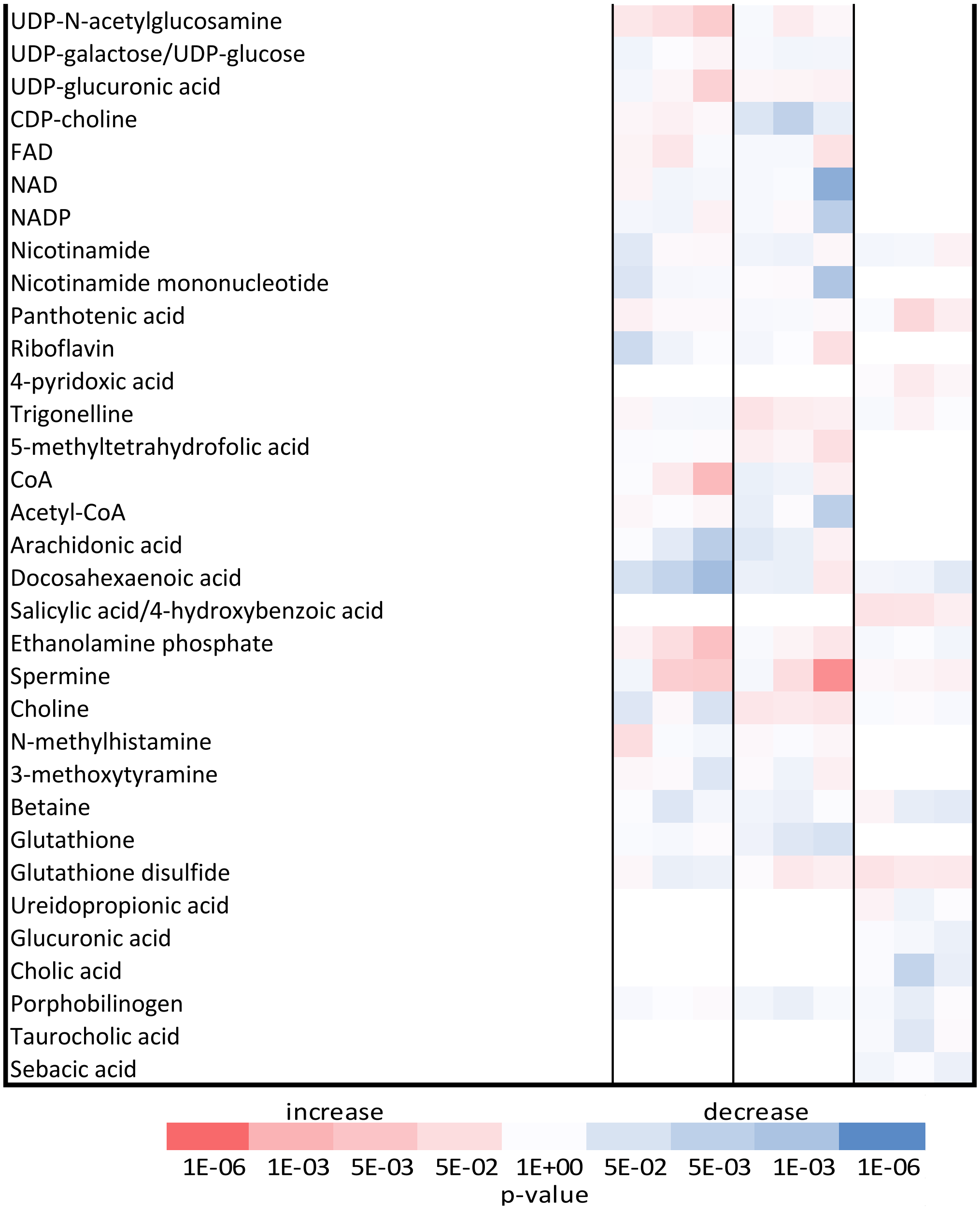
UDP-N-acetylglucosamine
UDP-galactose/UDP-glucose
UDP-glucuronic acid
CDP-choline
FAD
NAD
NADP
Nicotinamide
Nicotinamide mononucleotide
Panthotenic acid
Riboflavin
4-pyridoxic acid
Trigonelline
5-methyltetrahydrofolic acid
CoA
Acetyl-CoA
Arachidonic acid
Docosahexaenoic acid
Salicylic acid/4-hydroxybenzoic acid
Ethanolamine phosphate
Spermine
Choline
N-methylhistamine
3-methoxytyramine
Betaine
Glutathione
Glutathione disulfide
Ureidopropionic acid
Glucuronic acid
Cholic acid
Porphobilinogen
Taurocholic acid
Sebacic acid
increase
decrease
1E-06
1E-03
5E-03
5E-02
1E+00
5E-02
5E-03
1E-03
1E-06
p-value
