# Supplementary material for: Metabolomic and lipidomic changes triggered by lipopolysaccharide-induced systemic inflammation in transgenic APdE9 mice
Source: Sci Rep. 2021 Jun 22;11:13076. doi: 10.1038/s41598-021-92602-4 (PMC8219693; doi:10.1038/s41598-021-92602-4)
Supplement: Supplementary file 2 — Supplementary Information 2. [file 41598_2021_92602_MOESM2_ESM.docx]

**Metabolomic and lipidomic changes triggered by lipopolysaccharide-induced systemic inflammation in transgenic APdE9 mice**

*Elena Puris^1,2*†^,* *Štěpán Kouřil^3,4†^, Lukáš Najdekr^3^, Sanna Loppi^5,6^, Paula Korhonen^5^, Katja M. Kanninen^5^, Tarja Malm^5^, Jari Koistinaho^5,7^, David Friedecký^3,4^,*

*Mikko Gynther^1^*

*^1^School of Pharmacy, University of Eastern Finland, P.O. Box 1627, 70211 Kuopio, Finland*

*^2^Institute of Pharmacy and Molecular Biotechnology, Ruprecht-Karls-University, Im Neuenheimer Feld 329, 69120 Heidelberg, Germany (present address for E.P.)*

*^3^Institute of Molecular and Translational Medicine, Palacký University Olomouc, Hněvotínská 5, 77900 Olomouc, Czech Republic*

*^4^Department of Clinical Biochemistry, University Hospital Olomouc, I.P. Pavlova 6, 77900 Olomouc, Czech Republic*

*^5^A.I. Virtanen Institute for Molecular Sciences, University of Eastern Finland, P.O. Box 1627, 70211 Kuopio, Finland*

*^6^Department of Immunobiology, University of Arizona, 1656 E Mabel Street, Tucson, Arizona 85724-5221, USA (present address for S.L.)*

*^7^Neuroscience Center, Helsinki Institute for Life Science, University of Helsinki, Haartmaninkatu 8, 00290 Helsinki, Finland (present address for J.K.)*

^†^Elena Puris and Štěpán Kouřil should be considered joint first authors.

*****corresponding author: Elena Puris, Institute of Pharmacy and Molecular Biotechnology, Ruprecht-Karls-University, Im Neuenheimer Feld 329, 69120 Heidelberg, Germany; phone: +(358)449789164; email: elena.puris@uni-heidelberg.de

**Supplementary information 1 (Behavioural assessment - methods and results)**

**Methods**

The behavioral tests were performed in mice of WT plus LPS, APdE9, APdE9 plus LPS and WT control groups in the end of the second wash-out period (4 weeks after the last dose of LPS or saline).

*Novel Object Recognition test*

Novel Object Recognition test (NOR) measures both hippocampal and cortical damage affecting object recognition memory and interest towards novel object of the animal^1^. In this test, the mice from the investigated groups were placed to a novel, clean cage and let them to familiarize with the new environment for 10 min. 2 similar objects, wooden doorknobs, were placed in two corners of the cage. Time that the animal was sniffing or spending more than 5 s closer than 2 cm either of the objects was recorded using Ethovision program. Record time was 10 min. After this, animals were returned to their home cages. After 24 h of background recording, the animals were placed again to the test cage. This time, one object was again a wooden doorknob but the other one was replaced with a metallic one. Time that the animal was sniffing either object during 5 min time was recorded and the ratio between the time spend close to the novel and familiar object was calculated. The two measures, D1 and Discrimination Index (DI), were calculated. D1 is defined as the difference in exploration time for novel (T_N_) versus familiar objects (T_F_), i.e., the exploration time devoted to the novel object minus the time devoted to the familiar object, [D1 = (T_N_ − T_F_)]. The Discrimination Index (DI), allows discrimination between the novel and familiar objects, i.e., it uses the difference in exploration time for familiar object (T_F_), but then dividing this value by the total amount of exploration of the novel (T_N_) and familiar objects [DI = (T_N_ − T_F_)/(T_N_ + T_F_)]. DI value varies between +1 and −1, where a positive score indicates more time spent with the novel object, a negative score indicates more time spent with the familiar object, and a zero score indicates a null preference.

*Open Field Spontaneous Movement Activity using TruScan®*

Motor activity of the mice (exploration activity, moving in general, reaching, ambulatory and stereotypic movement) were measured with an automated activity monitor (TruScan®, Coulbourn Instruments, CO, USA) with infra-red-light beams. The test also measures the time animals spend in the center or in the margins of the box. In this test, mouse was placed to clean test box and its moving behavior was automatically measured by the TruScan® program for 10 min. Different parameters were then calculated such as ambulatory distance (cm), ambulatory movement time (s), number of stereotypic movements, time of stereotypic movements (s), time spent exploring the periphery of the arena (s) and time spent in the center of the arena (s). The measurement of ambulatory movement as an ambulatory distance and ambulatory movement time reflects total movements less stereotypy (the larger stereotypy if both are specified).

**Statistical analysis**

For the statistical analysis of the differences in parameters determined in behavioural tests between the groups, i.e. the WT plus LPS, APdE9, APdE9 plus LPS versus WT control, the one-way ANOVA followed by Dunnett's multiple comparisons test was used (GraphPad Prism 6, La Jolla, CA). Statistical significance was defined as *p* < 0.05.

**Results of behavioural tests**

*Novel Object Recognition test*

Using the NOR test, we evaluated changes in cognitive function induced in female wild-type (WT) mice treated with LPS, APdE9 mice with and without treatment with LPS versus WT control (Supplementary Fig. S1). The mean DI values in APdE9 mice, APdE9 plus LPS and WT plus LPS groups were negative indicating more time spent with the familiar object, while the mean DI for WT control mice was positive indicating more time spent with the novel object. There was statistically significant difference in D1 values in all investigated models compared to WT controls (Supplementary Fig. S1). These data suggest impaired cognitive function in all three models compared to WT controls.





**Supplementary Figure S1.** Results of Novel Object Recognition Test (NORT) in female wild-type (WT) mice treated with LPS (n = 9), APdE9 mice with (n = 9) and without (n = 9) treatment with LPS versus WT control (n = 11): (a) Discrimination Index (DI) and (b) D1 score (s). The data is presented as mean ± SD. Asterisks denote a statistically signiﬁcant diﬀerence from the respective control (**p* < 0.05, one-way ANOVA followed by Dunnett's multiple comparisons test).

*Open Field Spontaneous Movement Activity using TruScan®*

We analyzed the exploratory behavior and general locomotor activity in an open field arena. We observed a significantly greater locomotor activity in APdE9 mice treated with LPS as compared to WT control mice (Fig. S2). The ambulatory distance and time for ambulatory movement were significantly higher in APdE9 mice treated with LPS as compared to WT control mice (Fig. S2), while saline-treated APdE9 mice and LPS-treated WT mice did not possess significant changes in ambulatory distance and time for ambulatory movement. Other parameters, such as number of stereotypic movements, time spent for stereotypic movements, time spent exploring the periphery of the arena and time spent in the center of the arena did not differ between the investigated groups.

**

**

**Supplementary Figure S2.** Exploratory activity in female wild-type (WT) mice treated with LPS (n = 11), APdE9 mice with (n = 10) and without (n = 10) treatment with LPS versus WT control (n = 11) evaluated using TruScan® automated activity monitoring based on detection of breaks of infrared photobeams: (a) Ambulatory distance in sm; (b) Ambulatory movement time (s); (c) Number of stereotypic movements; (d) Time spent for stereotypic movements (s); (e) Time spent exploring the periphery of the arena (s); (f) Time spent in the center of the arena (s). The data is presented as mean ± SD. Asterisks denote a statistically signiﬁcant diﬀerence from the respective control (****p* < 0.005, one-way ANOVA followed by Dunnett's multiple comparisons test).

**References**

1 Antunes, M. & Biala, G. The novel object recognition memory: neurobiology, test procedure, and its modifications. *Cogn Process* **13**, 93-110, doi:10.1007/s10339-011-0430-z (2012).
